# Integrin-β6 Serves as a Potential Prognostic Serum Biomarker for Gastric Cancer

**DOI:** 10.3389/fonc.2021.770997

**Published:** 2021-11-02

**Authors:** Zequn Li, Yuqi Sun, Jianfei Xu, Hao Yang, Xiaodong Liu, Yulong Tian, Shougen Cao, Yanbing Zhou

**Affiliations:** Department of Gastrointestinal Surgery, The Affiliated Hospital of Qingdao University, Qingdao, China

**Keywords:** ITGB6, gastric cancer, serum biomarker, risk stratification, prognosis, liver metastasis

## Abstract

Discovering novel biomarkers that easily accessed is a key step towards the personalized medicine approach for gastric cancer patients. Integrin-β6 (ITGB6) is a subtype of integrin that is exclusively expressed on the surface of epithelial cells and is up-regulated in various tumors. In the present study, a retrospective cohort with 135 gastric cancer patients and a prospective cohort with 34 gastric cancer patients were constructed, ITGB6 expression were detected in both the serum specimens and the tissue specimens. Detailed clinicopathological parameters as well as patients’ survival were recorded. A nomogram including ITGB6 expression was also constructed and validated to predict the prognosis of gastric cancer patients. Results showed that serum ITGB6 expression was obviously increased and associated with tumor stage in gastric cancer patients, serum ITGB6 expression was relatively high in patients with liver metastasis. High ITGB6 expression indicated a poor prognosis, and nomogram including serum ITGB6 expression could predict the prognosis of gastric cancer patients effectively. Moreover, serum ITGB6 expression was associated with ITGB6 expression in tumor tissues. Furthermore, combined serum ITGB6 and CEA levels contributed to the risk stratification and prognostic prediction for gastric cancer patients. In addition, the serum expression of ITGB6 decreased significantly after radical surgery, and a new rise in serum ITGB6 expression indicated tumor recurrence or progression. The present study identified a novel serum biomarker for the risk stratification, prognostic prediction and surveillance of gastric cancer patients.

## Introduction

Gastric cancer is the fifth most commonly diagnosed cancer and the third leading cause of cancer-related death worldwide (1, 2). Advanced gastric cancer accounts for the majority in China, and radical surgical resection remains to be the most effective treatment strategy (3). The prognosis of advanced gastric cancer has improved dramatically over the recent decades due to the implement of novel surgical techniques, progression of chemotherapeutics and targeted drugs (4–6). However, the survival for patients with local or distant metastasis remains poor (7). Discovering easily accessible biomarkers, such as serum biomarkers, is urgently needed for optimizing patient care in patients with gastric cancer. At present, carcinoembryonic antigen (CEA) is one of the standard biomarkers for gastric cancer. However, it still exhibits low sensitivity and specificity (8).

Integrins are a family of heterodimeric cell membrane receptors that are expressed in most cells, where they mediate cell-cell and cell-extracellular matrix (ECM) interactions. Integrin β6 (ITGB6) was preliminarily identified in 1996 and it is exclusively expressed on epithelial cells during embryogenesis. ITGB6 expression also elevated during wound healing, fibrosis, and importantly, carcinogenesis (9, 10). As one of the key adhesion molecules on cell surface, ITGB6 was found to be involved in almost every step during tumor metastasis (11–14). Previous studies have demonstrated that ITGB6 was involved in the progression of gastric cancer. ITGB6 expression in gastric cancer tissues was closely associated with tumor stage, it also served as an independent prognostic indicator for the poor prognosis of gastric cancer. ITGB6 might be involved in the regulation of MMP expression, and contributed to tumor progression *via* ERK signaling (15–18). Nevertheless, the expression of serum ITGB6 in cancer is largely unknown so far.

Nomogram has been widely used in the prediction of cancer prognosis, which transforms traditional statistical predictive models into visualized probability estimates tailored to the needs of the individual patient. With continuous developments in molecular biology, applying biomarkers that reflect the malignant biological behaviors of tumors could potentially be a supplementary approach to the clinicopathological variables. Therefore, an effective and accurate model to predict the prognosis of gastric cancer is of vital importance for clinical-decision making.

The present study investigated whether serum ITGB6 level serves as a novel tumor biomarker for gastric cancer patients. Focusing on risk stratification, prognostic prediction and recurrence surveillance, we explored the clinical significance of serum ITGB6 levels for gastric cancer patients by both retrospective and prospective cohorts. This study provided a novel serum biomarker for gastric cancer patients, which might contribute to improving the prognosis and represent a key step forward towards the personalized medicine approach for advanced gastric cancer patients.

## Patients and Methods

### Bioinformatics Analysis

The ITGB6 mRNA expression data and corresponding clinical information for gastric cancer patients were obtained from The Cancer Genome Atlas (TCGA; https://tcga-data.nci.nih.gov/tcga/). Meanwhile, the differential mRNA expression level of ITGB6 between a variety of cancer tissues and normal ones were obtained using the TIMER database (https://cistrome.shinyapps.io/timer/). Related gene expression analysis and disease-free survival (DFS) from TCGA and GTEx databases were performed using the GEPIA database (http://gepia.cancerpku.cn/) (19).

### Retrospective Cohort

Between January 2017 and December 2017, we collected a total of 135 patients with gastric cancer that underwent surgical treatment at the Department of Gastrointestinal Surgery, the Affiliated Hospital of Qingdao University. The diagnosis was confirmed by routine pathological examination and inclusion criteria was as follows: (1) Serum samples with detailed clinicopathological data and medical records; (2) Postoperative survival time more than 1 month; (3) No history or signs of other malignancies. Follow-up data were recorded until June 2021, concerning survival time and progression of gastric cancer at the last visit. Tumor staging and histological classification were assessed according to the 8^th^ edition of the American Joint Committee on Cancer (AJCC) classification. The expression levels of serum CEA were routinely detected for all the enrolled patients at least one time in the medical record. Follow-up was conducted every 3-6 months and serum tumor biomarkers including CEA were measured. Imaging examination, including computed tomography (CT), magnetic resonance imaging (MRI) or positron emission tomographic scanning (PET-CT) were also selectively conducted for the evaluation of metastasis or recurrence. This study was approved by the Ethics Committee of the Affiliated Hospital of Qingdao University, China. Written informed consent was obtained from all the subjects.

### Prospective Cohort

A prospective study was carried out from Oct 2020 to Jun 2021, consisting of 34 patients with radical gastric cancer resection. Tumor tissues and corresponding adjacent normal tissues were obtained. None of the patients received chemotherapy or radiotherapy prior to surgery. Follow-up data was obtained until June 2021. Written informed consent was obtained from each patient, and this study was approved by the Ethics Committee of the Affiliated Hospital of Qingdao University, China. Registration of the prospective study was approved by the Chinese Clinical Trial Registry (ChiCTR1800018294).

### Serum ITGB6 and CEA Detection

Serum ITGB6 levels were detected using human ITGB6 ELISA kit (SEC099Hu, USCN Life Science Inc., Wuhan; China) according to manufacturer’s instructions. In brief, the tests were performed according to the instruction of the ELISA kit. Then added termination solution, incubated for 30 min in darkness (37 °C, 5% CO_2_) and measured absorbance value at 450nm. Serum CEA levels were determined using radioimmunoassay kits manufactured by Abbott Laboratories (Chicago, IL, USA). The cut-off point for serum ITGB6 expression was defined 0.5ng/ml, which was verified by the X-tile program.

### Nomogram Construction and Validation

Nomogram construction and validation were performed in accordance with the nomogram guidelines (20, 21). Univariate and multivariate Cox proportional hazard models were constructed to estimate the hazard ratios of prognostic factors and to evaluate independent prognostic risk factors. A nomogram was constructed according to the independent prognostic factors of survival. Besides, the prognosis of nomograms was realized by using the RMS software package in R software version 3.1.3 (https://www.r-project.org/). To assess the model performance, the discrimination and calibration of the nomogram were performed (22). The discriminative power of the nomogram was computed by Harrell’s concordance index (C-index) (23). The C-index ranges from 0.5-1.0, with 0.5 indicates the outcomes is no discrimination at all and 1.0 represents the perfect discrimination.

### Immunohistochemistry (IHC)

IHC was performed using paraffin-embedded tissue sections (4 μm), and protocol was previously described (24, 25). Briefly, the sections were dewaxed and hydrated, followed by antigen retrieval (in 0.01 mol/L citrate buffer solution, pH 6.0, heated to boiling for 2-3 min). Endogenous peroxidase was blocked with 3% H_2_O_2_. The sections were blocked by goat serum for 15 min and then immunostained with mouse antibody against ITGB6 (dilution 1:500, Biogen Idec, USA) at 4°C overnight. Secondary staining was performed with HRP-conjugated antibody using a MaxVision Kit and a 3, 5-diaminobenzidine (DAB) peroxidase substrate kit (Maixin Co, Fuzhou, China). The sections were then counterstained with hematoxylin, and representative images were obtained under an Olympus inverted microscope.

### Evaluation of Immunohistochemical Staining

Immunohistochemical staining was independently assessed by two experienced pathologists in a blinded manner. Staining was semi-quantitatively scored based on both the staining intensity (0, negative; 1, very weak; 2, weak; 3, moderate; 4, strong) and the percentage of positively stained cells (0, 0%; 1, 1%-25%; 2, 26%-50%; 3, 51%-75%; 4, 76%-100%). Both scores for each specimen were combined to obtain the final score of ITGB6 expression (ranging 0-8). The cut-off point for the sum of the scores was defined as follows, 0-5, low expression; 6-8, high expression, which was verified by the X-tile program.

### Statistical Analysis

All the statistical analyses were performed by SPSS 22.0 software (SPSS, Chicago, IL, USA). The association between ITGB6 expression and clinicopathological parameters were assessed by chi-square test or Fisher’s exact test. The receiver operating characteristic (ROC) curve analyses were conducted for the measurement of predictive accuracy index. The cumulative OS rates were calculated by Kaplan–Meier method, and the statistical differences between subgroups were calculated by log-rank test. Independent prognostic factors were identified by multivariate analysis with Cox-regression model. In cox regression analysis, cox model was built using forward stepwise method. *P* value < 0.05 was considered statistically significant.

## Results

### Serum ITGB6 Was a Potential Biomarker for Gastric Cancer That Associated With Tumor Stage

**Figure 1:**
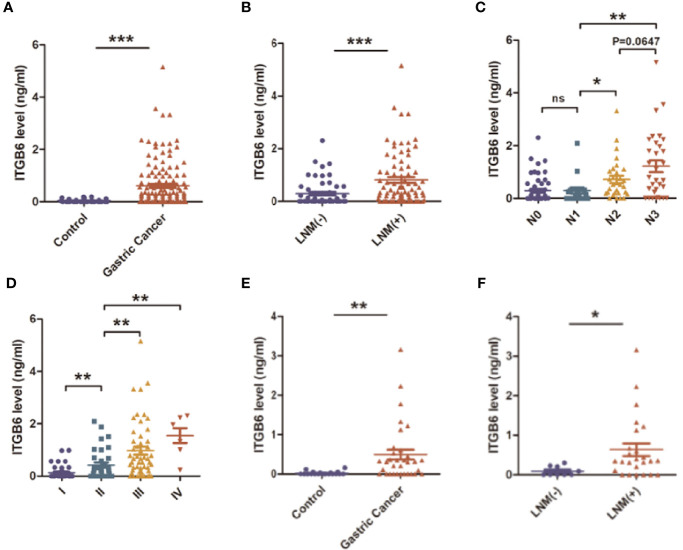
The expression and clinical significance of serum ITGB6 in gastric cancer. **(A)** The expression of serum ITGB6 in gastric cancer patients and healthy controls from a retrospective cohort. **(B)** The serum ITGB6 levels in gastric cancer patients with or without lymph node metastasis. **(C)** The serum ITGB6 levels in gastric cancer patients with different N stage from the retrospective cohort. **(D)** The serum ITGB6 levels in gastric cancer patients with different pathological stage from the retrospective cohort. **(E)** The expression of serum ITGB6 in gastric cancer patients and healthy controls from the prospective cohort. **(F)** The serum ITGB6 levels in gastric cancer patients with or without lymph node metastasis from the prospective cohort. ns, not statistically different; *P<0.05; **P<0.01; ***P<0.001.

To explore whether ITGB6 could be detected in the serum of gastric cancer patients, we investigated serum ITGB6 levels in a retrospective cohort containing 135 gastric cancer patients. The serum specimens of 32 healthy subjects that underwent physical health examination were used as control. Results showed that ITGB6 could be detected in the serum of gastric cancer patients, which ranged from 0-5.16ng/ml, and serum ITGB6 levels were significantly increased in gastric cancer patients (**Figure 1A**). Moreover, **Table 1** showed that increased ITGB6 serum levels were closely associated with the TNM stage of tumor size (*P*=0.001), T stage (*P*=0.050), N stage (*P*=0.001), TNM stage (*P*=0.007), neurovascular infiltration (*P*=0.008) and serum CEA levels (*P*<0.001). Importantly, ITGB6 levels gradually increased accompanied with advanced N stage and TNM stage (**Figures 1B–D**). Interestingly, serum ITGB6 expression was markedly increased in patients with liver metastasis (**Supplementary Figure 1**).

**Table 1:** Correlation between serum ITGB6 expression and clinical characteristics of patients with gastric cancer.

Characteristic	Number	Serum ITGB6 expression		χ^2^/t value	*P*-value
		Low (%)	High (%)		
Age (years)				0.263	0.608
≤60	70	45 (64.3)	25 (35.7)		
>60	65	39 (60.0)	26 (40.0)		
Gender				1.309	0.253
Male	104	62 (59.6)	42 (40.4)		
Female	31	22 (71.0)	9 (29.0)		
Tumor size				11.118	0.001^*^
<4	75	56 (74.7)	19 (25.3)		
≥4	60	28 (46.7)	32 (53.3)		
Tumor location				1.829	0.401
Cardia/Fundus	14	11 (78.6)	3 (21.34)		
Body	64	38 (59.4)	26 (40.6)		
Antrum/Pylorus	57	35 (61.4)	22 (38.6)		
Borrmann type				5.806	0.121
I	9	5 (55.6)	4 (44.4)		
II	86	48 (55.8)	38 (44.2)		
III	37	29 (78.4)	8 (21.6)		
IV	3	2 (66.7)	1 (33.3)		
T stage				7.827	0.050^*^
T1	28	22 (78.6)	6 (21.4)		
T2	27	19 (70.4)	8 (29.6)		
T3	59	34 (57.6)	25 (42.4)		
T4	21	9 (42.9)	12 (57.1)		
N stage				15.782	0.001^*^
N0	52	38 (73.1)	14 (26.9)		
N1	24	20 (83.3)	4 (16.7)		
N2	27	13 (48.1)	14 (51.9)		
N3	32	13 (40.6)	19 (59.4)		
AJCC pTNM stage^#^				7.202	0.007^*^
I-II	78	56 (71.8)	22 (28.3)		
III-IV	57	28 (49.1)	29 (50.9)		
Complications				1.058	0.304
Yes	17	13 (76.5)	4 (23.5)		
No	118	71 (60.2)	47 (39.8)		
Neurovascular infiltration				6.949	0.008^*^
Yes	90	49 (54.4)	41 (45.6)		
No	45	35 (77.8)	10 (22.2)		
CEA				16.683	<0.001^*^
Normal	85	64 (75.3)	21 (24.7)		
High	50	20 (40.0)	30 (60.0)		

^#^The 8^th^ AJCC classification criteria; *statistical difference.

To confirm our conclusion, we further explored the clinical significance of serum ITGB6 expression in a prospective cohort that consisting 34 subjects. Serum ITGB6 expression was also elevated in gastric cancer patients compared with those healthy volunteers (**Figure 1E**), and elevated serum ITGB6 expression was also associated with lymph node metastasis in the prospective cohort (**Figure 1F**). These results confirmed our previous findings that serum ITGB6 possibly serve as a potential biomarker for gastric cancer.

### High Serum ITGB6 Level Was Associated With Poor Survival of Gastric Cancer Patients

**Figure 2:**
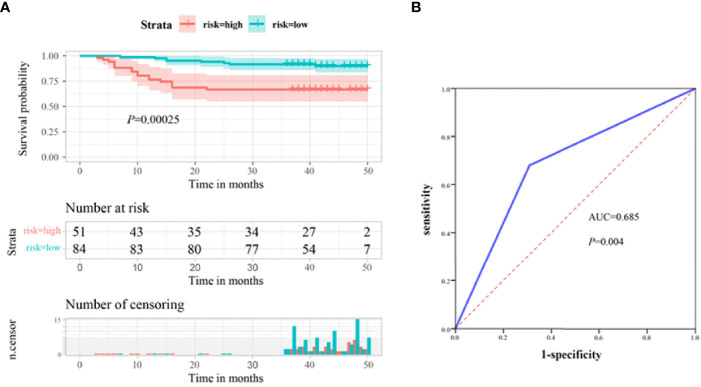
Serum ITGB6 level may serve as an unfavorable prognostic indicator for gastric cancer patients. **(A)** Survival analysis according to serum ITGB6 expression in a total number of 135 gastric cancer patients from a retrospective cohort. **(B)** ROC analysis was constructed for the prediction of prognosis of gastric cancer patients using serum ITGB6 expression.

As our previous results demonstrated that serum ITGB6 expression was closely associated with tumor progression in gastric cancer. Then we explored the prognostic value of serum ITGB6 expression in overall survival (OS) of gastric cancer patients. In the retrospective cohort, we followed the enrolled subjects for 3-50 months after surgery with a median follow-up period of 42 moths, and the overall survival (OS) of the patients was 81.5%. Results showed that the OS of gastric cancer patients with high expression of ITGB6 was 66.7%, which was lower than those patients with low expression of ITGB6 (90.5%) (**Figure 2A**). Moreover, TNM stage (*P*=0.002), neurovascular infiltration (*P*=0.049) and serum CEA levels (*P*<0.001) were also correlated with the prognosis of patients (**Table 2**).

**Table 2:** Univariate and multivariate Cox proportional hazard analyses of OS with gastric cancer patients.

	Univariate analysis		Multivariate analysis	
Variable	HR (95%CI)	*P-*value	HR (95%CI)	*P*-value
Sex (male *vs.* female)	0.423 (0.126-1.413)	0.162		
Age	1.171 (0.534-2.567)	0.694		
BMI	1.055 (0.954-1.168)	0.298		
Tumor size	1.007 (0.457-2.217)	0.987		
Tumor location		0.959		
Cardia/Fundus	Reference			
Body	0.851 (0.237-3.050)	0.804		
Antrum/Pylorus	0.936 (0.261-3.357)	0.920		
Borrmann type		0.415		
I	Reference			
II	0.605 (0.178-2.056)	0.421		
III	0.289 (0.065-1.293)	0.104		
IV	0.000 (0.000- ~)	0.981		
T stage		0.087		
T1	Reference			
T2	3.248 (0.338-31.225)	0.308		
T3	7.488 (0.984-56.966)	0.052		
T4	10.179 (1.252-82.779)	0.030^*^		
N stage		0.373		
N0	Reference			
N1	0.766 (0.203-2.889)	0.694		
N2	1.208 (0.395-3.691)	0.741		
N3	2.011 (0.775-5.217)	0.151		
AJCC pTNM stage	3.859 (1.611-9.248)	0.002^*^	2.599 (1.106-6.108)	0.029^*^
Complications	0.275 (0.037-2.030)	0.205		
Neurovascular infiltration	2.934 (1.007-8.552)	0.049^*^		
CEA	5.212 (2.174-12.494)	<0.001^*^	4.105 (1.692-9.961)	0.002^*^
ITGB6 expression	4.220 (1.820-9.789)	0.001^*^	3.138 (1.301-7.568)	0.011^*^

HR, hazard ratio; CI, confidence interval; *statistical difference.

Moreover, multivariate analysis was conducted to evaluate the independent prognostic factors for this cohort. Results demonstrated that high expression of ITGB6 (*P*=0.011), TNM stage (*P*=0.029) and high CEA levels (*P*=0.002) were independent unfavorable prognostic factors (**Table 2**). Furthermore, to evaluate the predictive value of ITGB6 on the prognosis of gastric cancer patients, time-dependent ROC analysis was conducted and the AUC was 0.685 (95% CI: 0.568-0.803, sensitivity: 69.1%, specificity: 68.0%) (**Figure 2B**). All these results verified that serum ITGB6 may serve as an unfavorable prognostic indicator for gastric cancer.

### Serum ITGB6 Expression Was Associated With ITGB6 Expression in Tumor Tissues

Previous research demonstrated that elevated ITGB6 in gastric cancer tissues might serve as a potential biomarker for tumor progression and the prognosis of patients. By analyzing TCGA and GTEx public available database, we found that the mRNA expression level of ITGB6 in gastric cancer was significantly increased compared with that in normal tissues (**Supplementary Figure 2A**). Moreover, Kaplan-Meier analysis revealed that ITGB6 high-expressed patients had relatively less-optimistic prognostic outcome in terms of DFS (**Supplementary Figure 2B**).

**Figure 3:**
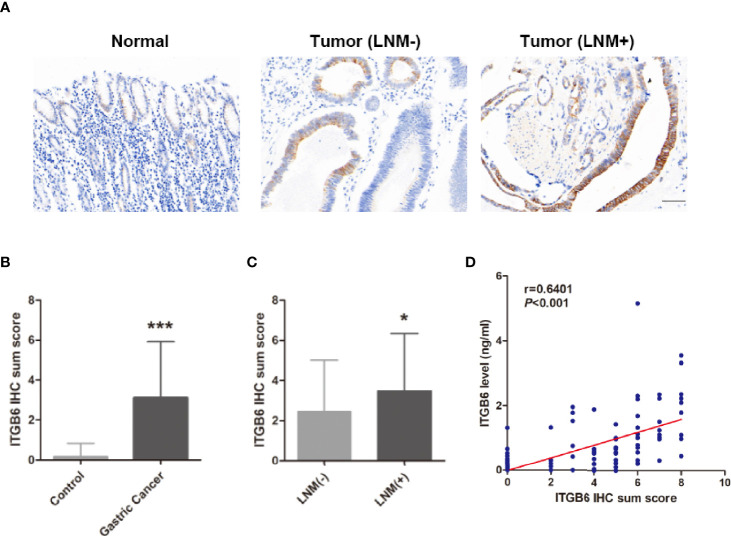
Serum ITGB6 expression was associated with ITGB6 expression in tumor tissues. **(A)** Representative IHC staining of ITGB6 in adjacent normal tissue, gastric cancer tissues with or without lymph node metastasis. (200×, scale bar=50μM) **(B)** IHC sum scores were used to evaluate ITGB6 expression in gastric cancer tissues. **(C)** IHC sum scores were used to evaluate ITGB6 expression in gastric cancer tissues with or without lymph node metastasis. **(D)** Correlation between tissue ITGB6 expression and serum ITGB6 expression. *P<0.05; ***P<0.001.

To explore whether serum ITGB6 expression was associated with ITGB6 expression in tumor tissues, we detected the expression of ITGB6 in gastric cancer tissues using IHC. Consistent with previous findings, elevated ITGB6 protein expression was also shown in gastric cancer tissues compared with adjacent normal tissues (**Figures 3A, B**). Moreover, ITGB6 expression in tumor tissues was also associated with tumor N stage (**Figure 3C**). Furthermore, ITGB6 expression in tumor tissues was correlated with serum ITGB6 levels in advanced gastric cancer (**Figure 3D**). All these results indicated that ITGB6 was present in both serum and tumor tissue of gastric cancer patients, and serum ITGB6 may serve as a potential biomarker for advanced gastric cancer.

### Construction and Validation of the Nomogram Based on Serum ITGB6 Level

To further assess how related clinicopathological parameters jointly impact on survival, the Cox regression model was used for univariate and multivariate survival analyses. Univariate analysis suggested that depth of invasion, TNM stage, neurovascular invasion, ITGB6 and CEA levels might be associated with the prognosis for gastric cancer patients (*P*<0.1). Subsequently, these variables were included in multivariate Cox proportional hazards analysis. Multivariate analysis confirmed that TNM stage, ITGB6 and CEA levels were independent prognostic factors for gastric cancer patients (**Table 2**).

**Figure 4:**
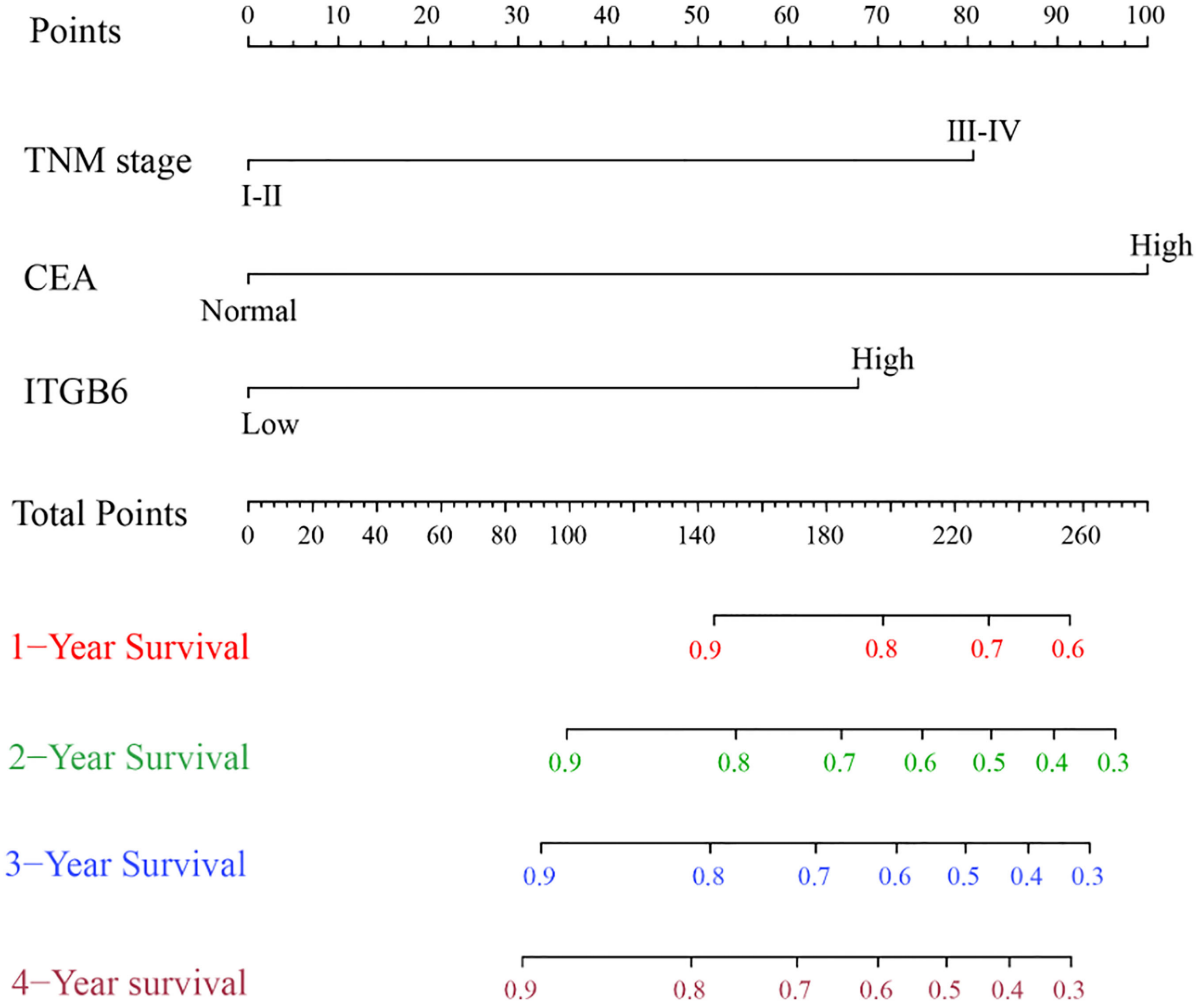
Prognostic Nomogram of 1-year, 2-year, 3-year and 4-year overall survival of 135 gastric cancer patients.

According to the aforementioned results, the TNM stage, serum ITGB6 and CEA expression were included in the final model to develop the nomogram for predicting overall survival (**Figure 4**). C-index was used to appraise the discrimination. The accuracy of this prediction model was relatively high, with a C-index of 0.792 (95% CI: 0.721-0.863).

### Combined Serum ITGB6 and CEA Levels Contributed to the Risk Stratification of Gastric Cancer

**Figure 5:**
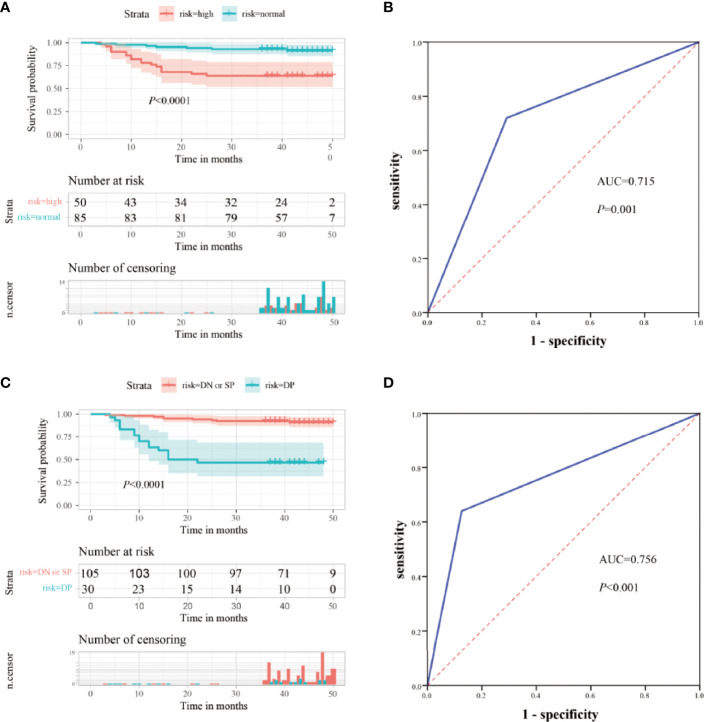
Clinical significance of combined serum ITGB6 and CEA levels for gastric cancer patients. **(A)** Survival analysis according to serum CEA levels in a total number of 135 gastric cancer patients from a retrospective cohort. **(B)** ROC analysis was constructed for the prediction of prognosis of gastric cancer patients using serum CEA expression. **(C)** Survival analysis according to combined serum ITGB6 and CEA levels in gastric cancer patient from the retrospective cohort. **(D)** ROC analysis was constructed for the prediction of prognosis of gastric cancer patients using combined serum ITGB6 and CEA expression.

CEA is commonly used for the risk stratification and recurrence follow up nowadays. Here we compared serum ITGB6 levels with serum CEA levels using both the retrospective cohort and the prospective cohort. Kaplan-Meier analysis showed that the OS of the normal preoperative CEA group was 91.8%, whereas that of the elevated preoperative CEA group was 64.0% (**Figure 5A**). Then the ROC analysis was used to evaluate the predictive prognostic performance of CEA, which led to an AUC=0.715 (95% CI: 0.601-0.828, sensitivity: 70.9%, specificity: 72.0%) (**Figure 5B**).

To enhance the specificity of predict patient survival, we evaluated the clinical values of combined biomarkers of CEA and ITGB6. Patients were divided into two subgroups based on the expression of CEA and ITGB6: double negative or single positive (n=105, 77.78%) and double positive (n = 30, 22.22%). Patients with double positive had OS rate of 46.7% compared with 91.4% for patients with double negative or single positive (**Figure 5C**). Moreover, the AUC for the combined two biomarkers reached to 0.756 (95% CI, 0.638-0.875), with an estimated sensitivity and specificity of 64.0% and 87.3% (**Figure 5D**). All these results indicated that combined ITGB6 and CEA levels may improve the specificity of predicting clinical outcomes of gastric cancer patients.

### Serum ITGB6 Served as a Potential Biomarker for Tumor Surveillance in Gastric Cancer Patients

**Figure 6:**
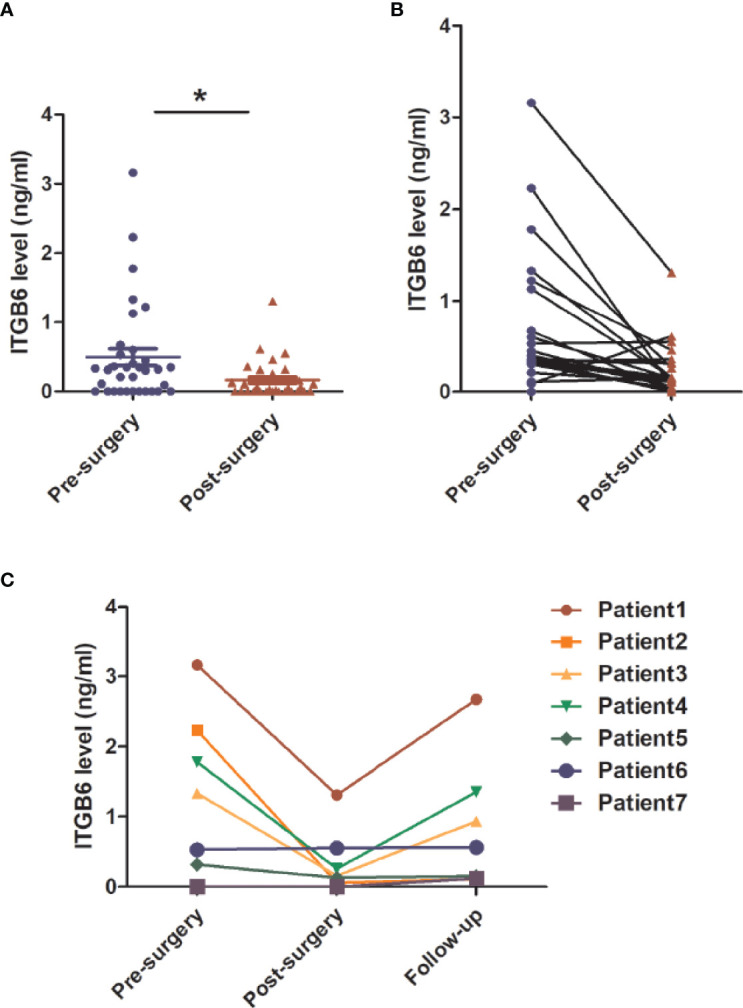
The application of serum ITGB6 level for tumor surveillance in gastric cancer patients from a prospective cohort. **(A, B)** Changes of serum ITGB6 levels after radical gastrectomy for gastric cancer patients from a prospective cohort. **(C)** Trend line of serum ITGB6 levels from 7 patients with tumor recurrence during follow-up. *P<0.05.

To investigate whether serum ITGB6 could be used for tumor surveillance and monitoring of tumor recurrence during the follow-up of gastric cancer patients, we detected the expression of serum ITGB6 before and after surgery, and followed up the patients for 6 months in the prospective cohort. Results showed that serum ITGB6 levels decreased dramatically after surgery for most of advanced gastric cancer patients (**Figures 6A, B**). Importantly, 7 patients presented recurrent disease during follow up. Among the patients with tumor recurrence, 4 of them had elevated serum ITGB6 levels, accompanied with increased tumor burden. One patient had sustained high serum ITGB6 level after surgery. In addition, serum ITGB6 was not detected both before and after surgery for a patient, but there was a slight elevation in serum ITGB6 level during tumor recurrence (**Figure 6C**). These results indicated that serum ITGB6 may serve as a biomarker for tumor surveillance of gastric cancer patients, but a cohort with larger sample size is also warranted.

## Discussion

Discovering easily accessible tumor biomarkers is crucial for optimizing patient care in patients with gastric cancer (26). Here we identified serum ITGB6 level may serve as a novel tumor biomarker for gastric cancer. ITGB6 was identified as an epithelial-specific expressed subtype of integrin that induced during wound healing, inflammation and carcinogenesis (10, 27). As a member of cell surface adhesion molecules, ITGB6 was involved in the tumorigenesis and progression of several tumors. It has been demonstrated that ITGB6 participated in almost every step during tumor metastasis. The expression of ITGB6 in gastric cancer tissues was associated with matrix metalloproteinase 9 (MMP-9), and ITGB6 might participate in the invasiveness of gastric cancer as a downstream effector of vascular endothelial growth factor (VEGF), indicating that ITGB6 is a key molecule that involved in the invasiveness and metastatic potential of gastric cancer (14–17). Consistent with these findings, based on both retrospective and prospective cohorts, the present study found that high baseline serum ITGB6 levels were observed in patients with advanced gastric cancer, especially patients with lymph node metastasis or distant metastasis. Previous study also demonstrated that integrin αvβ6 may contribute to targeted liver metastasis of colorectal cancer *via* the SDF-1/CXCR4 axis (28), and we should notice the phenomenon that serum ITGB6 expression was markedly increased in patients with liver metastasis. Although the sample size was limited, further research focusing on the role of ITGB6 in liver metastasis of gastric cancer is warranted. The present study demonstrated that patients with a serum ITGB6 level >0.5ng/ml were highly suspected to have lymph node metastasis or distant metastasis, indicating that ITGB6 might serve as a potential marker for the risk stratification of gastric cancer patients.

ITGB6 was considered to be a prognostic indicator as its increased expression in tumor tissues was significantly associated with the prognosis of patients in various tumors (29–34). It has been demonstrated that positive ITGB6 expression in gastric cancer tissues was linked to significantly reduced survival times (15, 17). Importantly, our results showed that serum ITGB6 levels were closely associated with unfavorable prognosis of patients with gastric cancer. Based on the aforementioned findings, a nomogram for predicting the overall survival for gastric cancer patients based on ITGB6 expression was established, which showed a favorable predictive efficacy.

As one of the standard biomarker for gastric cancer, CEA is commonly used for the risk stratification and recurrence follow up nowadays (3, 35). The present study also showed that serum ITGB6 levels was associated with CEA expression for gastric cancer patients. Moreover, combined serum ITGB6 and CEA levels significantly improved the efficacy for the risk stratification of gastric cancer.

Importantly, the present study also constructed a prospective cohort for confirmation of the conclusion, as well as exploring the clinical significance of serum ITGB6 levels during follow-up. A dramatic decrease of serum ITGB6 expression showed after surgery for most of the enrolled subjects, indicating that serum ITGB6 levels may be associated with tumor burden for gastric cancer patients. Further results also preliminary confirmed that serum ITGB6 levels might serve as a potential biomarker for tumor surveillance and monitoring of tumor recurrence during follow up. Rebounded high serum ITGB6 expression may indicate tumor recurrence and a sustained high serum ITGB6 level might represent poor prognosis.

Most of previous research mainly focus on tissue-expressed ITGB6 and as far as we know, only one study detected serum ITGB6 expression in patients with colorectal cancer, which revealed that serum ITGB6 may serve as a potential biomarker for diagnosis and surveillance of colorectal cancer (14, 36–38). For the first time, we demonstrated that serum ITGB6 level may serve as an effective biomarker for the risk stratification and prognostic prediction of gastric cancer patients. Interestingly, we should notice that serum ITGB6 expression was associated with ITGB6 expression in tumor tissues, and as an easily accessed specimen, serum is undoubtedly more convenient and more likely to be accepted during the perioperative period and follow-up visit, which represents a better applicative prospect. In order to evaluate the risk stratification and predict the prognosis of gastric cancer patients, we strongly recommend a routine serum ITGB6 level detection within the perioperative period and during follow-up.

A limitation of this study is the limited sample size for both the retrospective and the prospective cohort. And the follow-up results of the prospective cohort with larger size is also warranted. Moreover, as more and more gastric cancer patients receive chemotherapy before or after surgery nowadays, the effect of chemotherapy on both serum and tissue ITGB6 expression is also deserved further investigation. In addition, a multicenter prospective cohort study is also needed in the further research.

In conclusion, the present study identified a novel potential serum biomarker for the risk stratification, prognostic prediction and recurrence surveillance for gastric cancer, which deserves further validation and application. And such easily accessed biomarker might essentially contribute to an optimized patient care for patients with gastric cancer.

## Data Availability Statement

The raw data supporting the conclusions of this article will be made available by the authors, without undue reservation.

## Ethics Statement

The studies involving human participants were reviewed and approved by the Ethics Committee of the Affiliated Hospital of Qingdao University. The patients/participants provided their written informed consent to participate in this study. Written informed consent was obtained from the individual(s) for the publication of any potentially identifiable images or data included in this article.

## Author Contributions

YZ, SC, and ZL designed the study. YS performed the experiments. JX and HY contributed to data analyses. XL and YT collected tissue specimens and clinical data. ZL and YS wrote the paper. YZ and SC revised the paper. All authors contributed to the article and approved the submitted version.

## Funding

This work was supported by Shandong Provincial Natural Science Foundation, China (No. ZR2020QH225), Key Research Project of Shandong Province, China (2016GGB01022), Qingdao Minsheng Science and Technology Foundation, Shandong, China (No. 14-2-3-5-nsh) and Qingdao Science and Technology Plan Project (No. 13-1-4-220-jch).

## Conflict of Interest

The authors declare that the research was conducted in the absence of any commercial or financial relationships that could be construed as a potential conflict of interest.

## Publisher’s Note

All claims expressed in this article are solely those of the authors and do not necessarily represent those of their affiliated organizations, or those of the publisher, the editors and the reviewers. Any product that may be evaluated in this article, or claim that may be made by its manufacturer, is not guaranteed or endorsed by the publisher.
