# Supplementary material for: Integrin-β6 Serves as a Potential Prognostic Serum Biomarker for Gastric Cancer
Source: Front Oncol. 2021 Nov 2;11:770997. doi: 10.3389/fonc.2021.770997 (PMC8593195; doi:10.3389/fonc.2021.770997)
Supplement: Supplementary file 1 [file DataSheet_1.docx]

**Supplementary Figure 1**


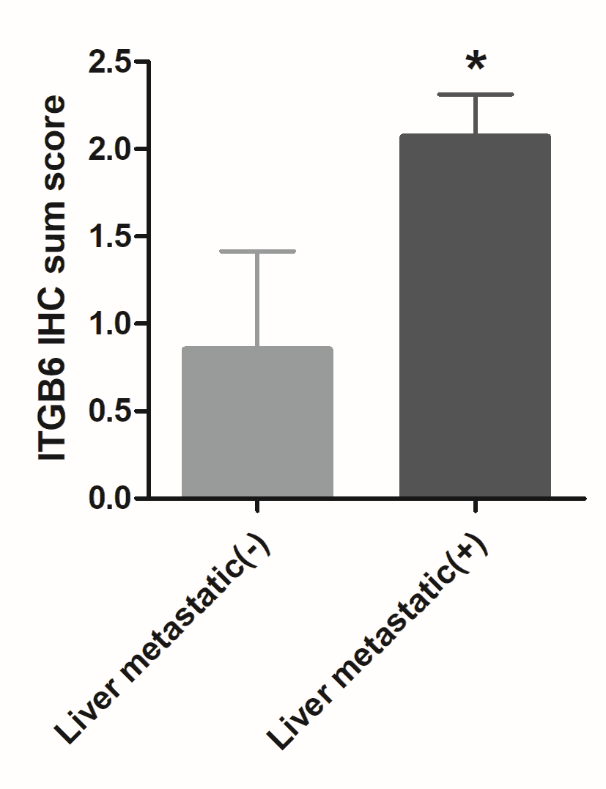


**Supplementary Figure 1. Serum ITGB6 expression in stage IV gastric cancer patients with or without liver metastasis.**

**Supplementary Figure 2**


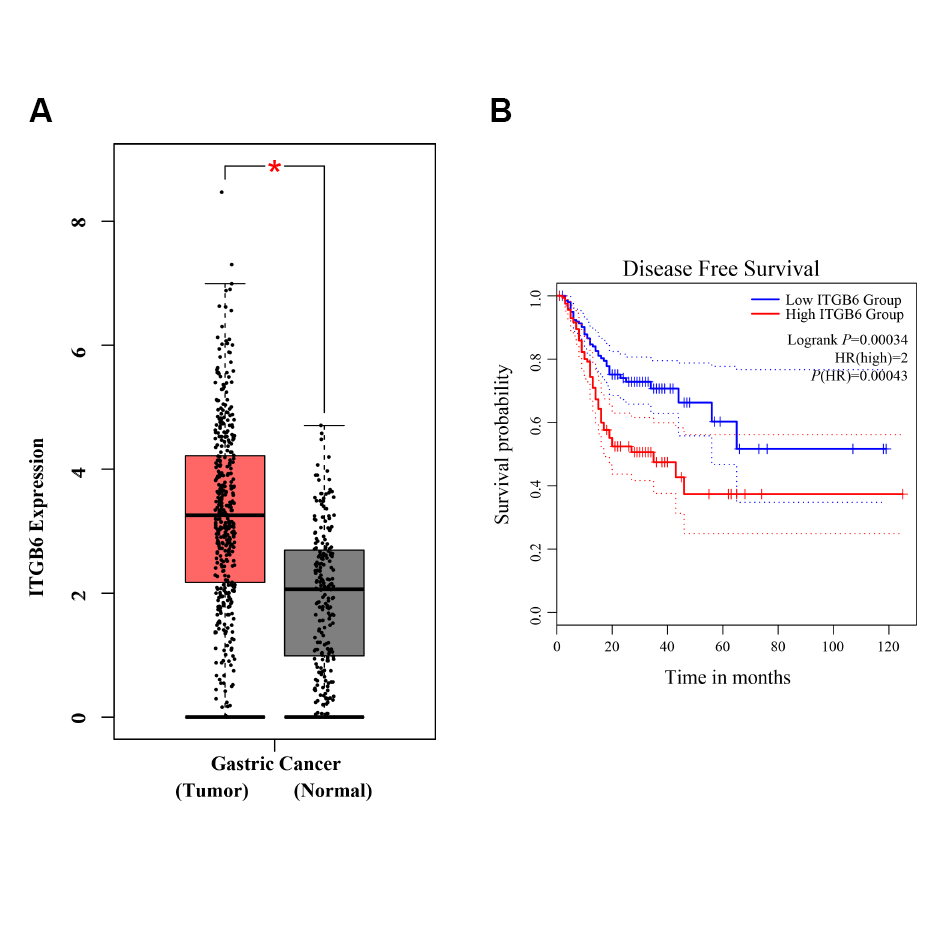


**Supplementary Figure 2. Clinical significance of ITGB6 expression in gastric cancer tissues.**

1. The expression of ITGB6 in gastric cancer tissues and normal tissues at mRNA levels.
2. Kaplan-Meier analysis according to tissue ITGB6 expression from the TCGA database.
